# Improved Immunodetection of Endogenous α-Synuclein

**DOI:** 10.1371/journal.pone.0023939

**Published:** 2011-08-19

**Authors:** Byung Rho Lee, Tetsu Kamitani

**Affiliations:** Department of Medicine, Center for Molecular Chaperone/Radiobiology and Cancer Virology, Georgia Health Sciences University, Augusta, Georgia, United States of America; Brigham and Women's Hospital, Harvard Medical School, United States of America

## Abstract

α-Synuclein is a key molecule in understanding the pathogenesis of neurodegenerative α-synucleinopathies such as Parkinson's disease. Despite extensive research, however, its precise function remains unclear partly because of a difficulty in immunoblotting detection of endogenous α-synuclein. This difficulty has largely restricted the progress for α-synucleinopathy research. Here, we report that α-synuclein monomers tend to easily detach from blotted membranes, resulting in no or very poor detection. To prevent this detachment, a mild fixation of blotted membranes with paraformaldehyde was applied to the immunoblotting method. Amazingly, this fixation led to clear and strong detection of endogenous α-synuclein, which has been undetectable by a conventional immunoblotting method. Specifically, we were able to detect endogenous α-synuclein in various human cell lines, including SH-SY5Y, HEK293, HL60, HeLa, K562, A375, and Daoy, and a mouse cell line B16 as well as in several mouse tissues such as the spleen and kidney. Moreover, it should be noted that we could clearly detect endogenous α-synuclein phosphorylated at Ser-129 in several human cell lines. Thus, in some tissues and cultured cells, endogenous α-synuclein becomes easily detectable by simply fixing the blotted membranes. This improved immunoblotting method will allow us to detect previously undetectable endogenous α-synuclein, thereby facilitating α-synuclein research.

## Introduction

α-Synuclein (α-syn) is a small soluble protein (17 kDa) that contains 140 amino acids and is predominantly expressed in the brain. α-Syn localizes to synaptic vesicles and the nucleus—hence the name “synuclein” [1], [2]. Although the exact function of α-syn is still unclear, substantial evidence now exists to suggest that this protein is predominantly natively unfolded in solution but can bind to phospholipid membranes by adopting an α-helical secondary structure in its N-terminal region [3], [4]. In neurons, α-syn regulates the pool of synaptic vesicles through an interaction with membranes [2].

Clinically, α-syn is a well-known molecule because mutations and copy number variation in the α-syn gene have been linked to Parkinson's disease (PD) [5], [6], [7], [8]. Furthermore, inclusions containing α-syn (called Lewy bodies) are pathological hallmarks of PD [9], [10]. PD is the second most common neurodegenerative disease and is characterized by the loss of dopaminergic neurons in the substantia nigra. The loss appears to be caused by neuronal cell death due to toxicity of accumulated α-syn [2]. Thus, α-syn is a central molecule in understanding the development of PD. Much effort has been directed toward investigating the α-syn molecule biochemically as well as immunohistochemically.

However, the application of immunoblotting to analyze the expression of endogenous α-syn, especially in cultured cells, has been limited [11]. This is partly because appropriate antibodies against α-syn have not been available until recently. Second, α-syn may not be transferred or immobilized efficiently on blotting membranes. Indeed, many researchers have experienced difficulty in detecting endogenous α-syn in cultured cells using a conventional immunoblotting method [11]. Interestingly, a similar problem was previously reported in immunodetection of human hemoglobin chains. Recently, however, this problem was solved by fixing the proteins onto blotted membranes with paraformaldehyde (PFA) at low concentration [12]. To improve immunodetection of endogenous α-syn, we applied this fixation method to our immunoblotting, resulting in clear and strong detection of endogenous α-syn. Here, we demonstrate the improved immunodetection method and reevaluate the expression levels of endogenous α-syn in cell lines and tissues. Furthermore, we show that this method is more suitable to studying the ratio of α-syn aggregates to its monomers.

## Materials and Methods

### Cell Lines and Culture Conditions

BJAB and U251MG were generous gifts from Dr. Fred Wang (Harvard Medical School) and Dr. Yoshiki Saito (M. D. Anderson Cancer Center), respectively. Other cell lines were purchased from American Type Culture Collection (Manassas, VA). K562, HL60, U937, Y79 and BJAB cells were cultured in RPMI 1640 medium supplemented with 10% fetal calf serum and antibiotics. Other cell lines were maintained in Dulbecco's modified Eagle's medium supplemented with 10% fetal calf serum and antibiotics.

### Mouse tissues

Tissue lysates were prepared from C57BL/6J mice, which were purchased from Jackson Laboratory (Bar Harbor, ME). These mice were maintained in a facility and program accredited by the Association for Assessment and Accreditation of Laboratory Animal Care International (AAALAC) with the approval number A3307-01. The animal use protocol (#BR08-09-103) was authorized by the Institutional Animal Care and Use Committee (IACUC) of Georgia Health Sciences University on September 18, 2008.

### Immunostaining

Cells were cultured on a coverslip in a 3.5-cm dish. After 24 h, the cells were fixed in a 4% PFA solution for 20 min, stained with 0.1 µg/ml of 4′,6-diamidine-2′-phenylindole dihydrochloride (DAPI; Roche Diagnostics, Indianapolis, IN) for 10 min, and permeabilized with 0.1% TritonX-100 for 10 min at room temperature. After blocking with 5% horse serum, the cells were first labeled with mouse monoclonal anti-α-syn (4D6) antibody (dilution 1∶1,000) overnight at 4°C. After washing, the slides were labeled with Alexa fluor 488-conjugated anti-mouse IgG (Molecular Probes, Eugene, OR) (dilution 1∶1,000) for 1 h at room temperature. Finally, the slides were analyzed by an Axio Imager M1 microscope (Zeiss). The localization of α-syn was shown by the green fluorescence of Alexa fluor 488. The nuclei were shown by the blue fluorescence of DAPI.

### Immunoblotting

Protein samples were treated for 30 min at 50°C in a sample-treating solution containing 2% SDS and 5% β-mercaptoethanol. The samples were then loaded onto a 12% or 15% SDS-polyacrylamide gel (7.0×8.3 cm ×0.75 mm) and electrophoresed using Mini-Protean Tetra system (Bio-Rad) at 15 mA (current constant). After electrophoresis, proteins separated on the gel were transferred onto a methanol-activated PVDF membrane (Immobilon-P, pore size 0.45 µm, Millipore) or a nitrocellulose membrane (Protran BA85, pore size 0.45 µm, Whatman) for 2 h using TE22 Mighty Small Transfer system (Hoefer Scientific) at 100 V (voltage constant). The membrane was then treated with or without phosphate-buffered saline (PBS) containing 0.4% PFA for 30 min at room temperature, followed by blocking for 1 h with 5% skim milk (Carnation) in Tris-buffered saline containing 0.1% Tween-20 (TBS-T). The membrane was then incubated for 1 h with a primary antibody in TBS-T containing 1% skim milk. As primary antibody, mouse monoclonal anti-α-syn antibodies 4D6 and LB509 (Santa Cruz Biotechnology) and rabbit monoclonal anti-phospho α-syn antibody EP1536Y (Epitomics, Burlingame, CA) were used at a dilution 1∶1,000, and rabbit polyclonal anti-actin antibody (Sigma) was also used at a dilution 1∶5,000. After washing with TBS-T containing 1% skim milk for 5 min three times, the membrane was incubated for 1 h with a secondary antibody, horseradish peroxidase-conjugated anti-mouse IgG or anti-rabbit IgG antibody (Santa Cruz Biotechnology), in TBS-T containing 1% skim milk. After washing with TBS-T for 10 min three times, protein bands on the membrane were detected by chemiluminescence method using ECL-Plus immunoblotting detection system (GE Healthcare).

### Generation of α-Syn-knockdown Cell Line

We knocked down endogenous α-syn by RNAi to establish an α-syn-knockdown cell line of human melanoma SK-MEL28 cells. In brief, we used lentivirus-mediated RNAi, called Mission® short hairpin RNA (shRNA) system (Sigma-Aldrich). According to the protocol provided with the system, lentiviruses were generated and infected to SK-MEL28 cells. Infected cells were then selected with puromycin.

### Recombinant α-Syn Protein

To investigate α-syn monomers and its aggregates, we purchased recombinant human α-syn protein (Cat. #S7820, Sigma-Aldrich) that was expressed in *E. coli* and purified.

## Results

### Discrepancy between Immunoblotting and Immunostaining in α-Syn Detection

**Figure 1:**
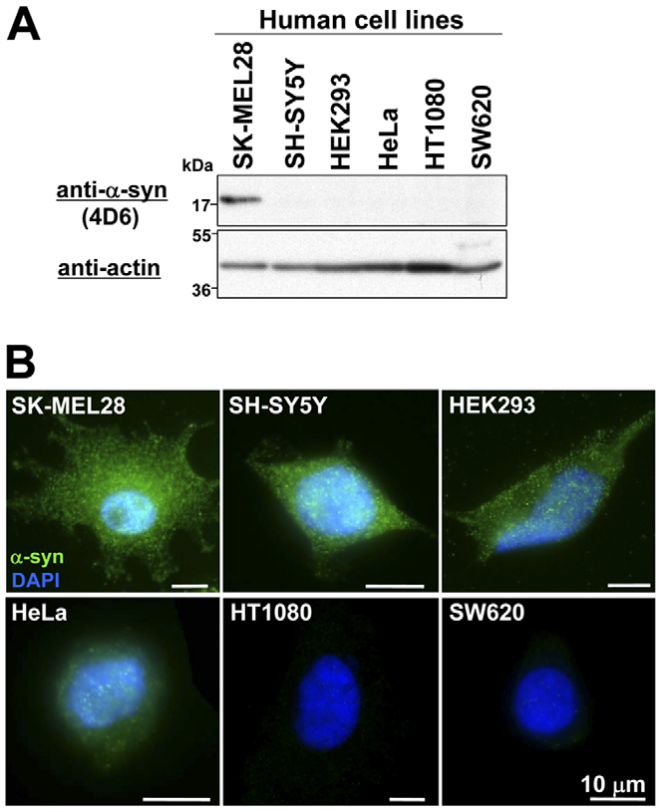
Detection of endogenous α-syn. ***A***, Conventional immunoblotting of various human cell lines. Total cell lysates were prepared from 6 human cell lines: SK-MEL28 (malignant melanoma), SH-SY5Y (neuroblastoma), HEK293 (human embryonic kidney cell), HeLa (cervical adenocarcinoma), HT1080 (lung fibrosarcoma), and SW620 (colon adenocarcinoma). The lysates (3 µl) containing ∼10 µg proteins were loaded per lane onto a 15% SDS-polyacrylamide gel. After SDS-PAGE and Western transfer onto a PVDF membrane, immunoblotting was performed using anti-α-syn antibody 4D6 (*upper panel*). To demonstrate near equal loading amount of total cell lysates, immunoblotting using anti-actin antibody was also performed (*lower panel*). Molecular size markers are shown in kilodaltons (kDa). ***B***, α-Syn immunostaining of various human cell lines. The indicated cell lines were fixed with PFA, stained with DAPI, permeabilized with 0.1% TritonX-100, and immunostained with mouse monoclonal anti-α-syn antibody 4D6. After washing, cells were labeled with Alexa Fluor 488-conjugated goat anti-mouse IgG antibody. The cells were then analyzed by fluorescence microscopy. The localization of endogenous α-syn is shown by the green fluorescence of Alexa Fluor 488. Nuclear counterstaining is shown by the blue fluorescence of DAPI. Scale bar indicates 10 µm.

Although α-syn is predominantly expressed in nervous system tissues [1], it is difficult to detect endogenous α-syn in cultured cell lines by immunoblotting. Indeed, we recently demonstrated that endogenous α-syn is undetectable in human neuroblastoma SH-SY5Y cells, but detectable in human melanoma cells such as SK-MEL28 [11]. To further investigate endogenous α-syn, we performed immunoblotting and immunostaining using 6 human cell lines: SK-MEL28 (malignant melanoma), SH-SY5Y (neuroblastoma), HEK293 (human embryonic kidney cell), HeLa (cervical adenocarcinoma), HT1080 (lung fibrosarcoma), and SW620 (colon adenocarcinoma). As shown in Fig. 1A, endogenous α-syn is detectable only in SK-MEL28, whereas it was undetectable in other human cell lines, which is consistent with our recent report [11].

Next, using an indirect immunofluorescent staining method, we immunostained endogenous α-syn in the same cell lines with the same antibody (4D6) at the same dilution. As shown in Fig. 1B, endogenous α-syn was clearly immunostained in SK-MEL28, SH-SY5Y, HEK293, and HeLa cells, but not in HT1080 and SW620 cells. The staining was strong in SK-MEL28, SH-SY5Y, and HEK293 cells, while it was weak in HeLa cells. To confirm these results, we then immunostained the same cell lines with another anti-α-syn antibody. The staining results were the same as those of 4D6 (data not shown).

In general, the indirect immunofluorescent staining is not a highly sensitive method. In our experiments, however, this method allows us to detect endogenous α-syn more sensitively than immunoblotting, suggesting that there is an unknown problem in a conventional method of immunoblotting for α-syn detection.

### Improved Method of Immunoblotting for α-Syn Detection

**Figure 2:**
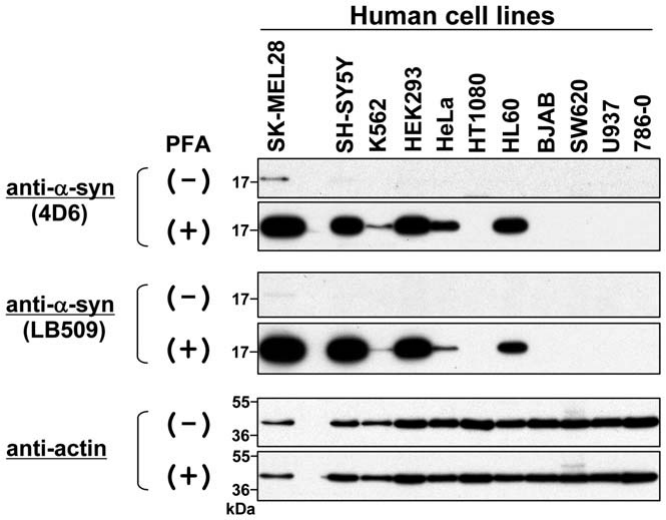
Immunoblotting of endogenous α-syn in various human cell lines. Total cell lysates were prepared from 11 human cell lines: SK-MEL28 (malignant melanoma), SH-SY5Y (neuroblastoma), K562 (erythroleukemia), HEK293 (human embryonic kidney cell), HeLa (cervical adenocarcinoma), HT1080 (lung fibrosarcoma), HL60 (promyelocytic leukemia), BJAB (Burkitt's lymphoma), SW620 (colon adenocarcinoma), U937 (myeloid leukemia), and 786-0 (renal cell carcinoma). The protein samples (∼10 µg) were loaded onto a 15% SDS-polyacrylamide gel and electrophoresed, followed by Western transfer onto a PVDF membrane. The membrane was then fixed with or without 0.4% PFA for 30 min. Afterwards, the membrane was blocked with 5% skim milk and incubated with a primary antibody such as anti-α-syn antibody 4D6, anti-α-syn antibody LB509, or anti-actin antibody. The membrane was then incubated with a secondary antibody conjugated with horseradish peroxidase. Protein bands on the membrane were detected by ECL-Plus detection system. Symbols (+) and (−) indicate presence and absence of membrane fixation with 0.4% PFA, respectively. Molecular size markers are shown in kilodaltons (kDa).

Previously, Suzuki, *et al.* reported that human hemoglobin can be efficiently detected by immunoblotting when blotted membranes are fixed with 0.4% PFA [12]. To improve our method for α-syn detection, we applied this membrane fixation to our immunoblotting. First, we performed immunoblotting with or without membrane fixation to detect endogenous α-syn in 11 human cell lines: SK-MEL28, SH-SY5Y, K562 (erythroleukemia), HEK293, HeLa, HT1080, HL60 (promyelocytic leukemia), BJAB (Burkitt's lymphoma), SW620, U937 (myeloid leukemia), and 786-0 (renal cell carcinoma). As shown in Fig. 2, when a conventional method (without membrane fixation) was used, endogenous α-syn was weakly detected only in SK-MEL28 cells, but not in the others (see PFA (−)). However, when an immunoblotting method with membrane fixation was used, endogenous α-syn was strongly detected in SK-MEL28, SH-SY5Y, HEK293, and HL60 cells (see PFA (+)). In addition, it was weakly, but clearly, detected in K562 and HeLa cells. Importantly, the expression levels of endogenous α-syn in SK-MEL28, SH-SY5Y, HEK293, HeLa, HT1080, and SW620 cells corresponded to those detected by immunostaining (see Fig. 2 and Fig. 1B).

### Reevaluation of Expression Levels of Endogenous α-Syn in Cell Lines and Tissues

**Figure 3:**
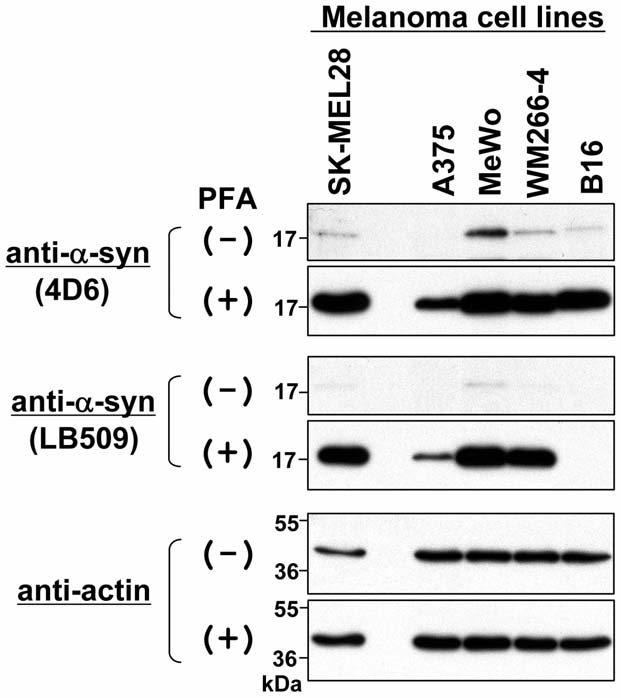
Immunoblotting of endogenous α-syn in various melanoma cell lines. Total cell lysates were prepared from 4 human melanoma cell lines (SK-MEL28, A375, MeWo, and WM266-4) and 1 mouse melanoma cell line (B16). The protein samples (∼10 µg) were loaded onto a 15% SDS-polyacrylamide gel and electrophoresed, followed by Western transfer onto a PVDF membrane. The membrane was then fixed with or without 0.4% PFA for 30 min. Afterwards, the membrane was blocked and incubated with a primary antibody (anti-α-syn antibody 4D6, anti-human α-syn antibody LB509, or anti-actin antibody) and a secondary antibody conjugated with horseradish peroxidase. Protein bands on the membrane were detected by ECL-Plus detection system. Symbols (+) and (−) indicate presence and absence of membrane fixation with 0.4% PFA, respectively. Molecular size markers are shown in kilodaltons (kDa).

Next, we reevaluated expression levels of endogenous α-syn in cell lines that we examined in our recent publication [11]. Fig. 3 shows the expression levels of endogenous α-syn in various melanoma cell lines. Although endogenous α-syn was undetectable in A375 using a conventional method (see PFA (−)), PFA fixation allowed us to detect it by 4D6 in all tested cell lines such as human melanoma SK-MEL28, A375, MeWo, and WM266-4 and mouse melanoma B16 (see PFA (+)). It should be noted that mouse α-syn in B16 was detected by 4D6, but not by LB509, which reacts with human α-syn only [13]. This result suggests that the mouse α-syn band detected by 4D6 on a PFA-fixed membrane is not a non-specific band.

**Figure 4:**
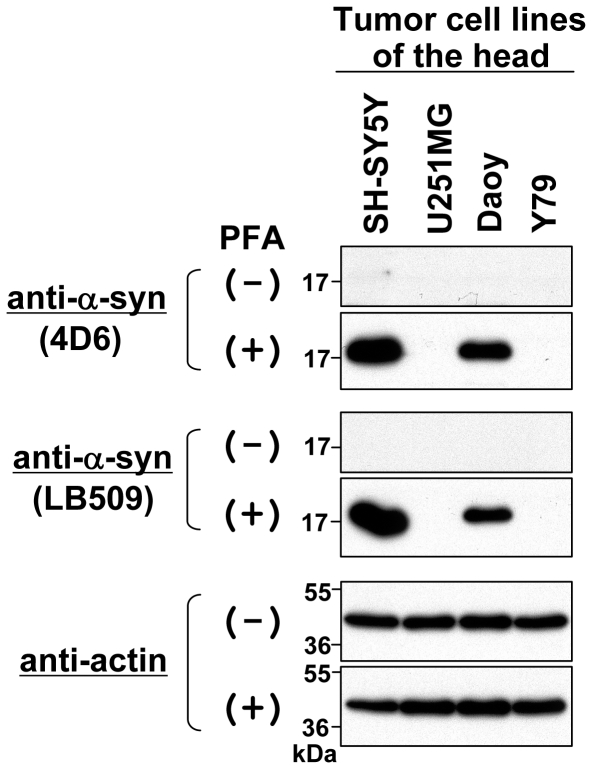
Immunoblotting of endogenous α-syn in tumor cell lines of the head. Total cell lysates were prepared from human retinoblastoma cell line Y79 and 3 human brain tumor cell lines: SH-SY5Y (neuroblastoma), U251MG (glioblastoma), and Daoy (medulloblastoma). The protein samples (∼10 µg) were loaded onto a 15% SDS-polyacrylamide gel and electrophoresed, followed by Western transfer onto a PVDF membrane. The membrane was then fixed with or without 0.4% PFA for 30 min. Afterwards, the membrane was blocked and incubated with a primary antibody (anti-α-syn antibody 4D6, anti-α-syn antibody LB509, or anti-actin antibody) and a secondary antibody conjugated with horseradish peroxidase. Protein bands on the membrane were detected by ECL-Plus detection system. Symbols (+) and (−) indicate presence and absence of membrane fixation with 0.4% PFA, respectively. Molecular size markers are shown in kilodaltons (kDa).

Since α-syn is predominantly expressed in the nervous system [1], we also examined its expression levels in human cell lines derived from the nervous system, including the retinoblastoma Y79 cell line and 3 brain tumor cell lines, SH-SY5Y, U251MG (glioblastoma), and Daoy (medulloblastoma). As shown in Fig. 4, when a conventional method of immunoblotting was used, the expression of α-syn was undetectable in the brain tumor and retinoblastoma cell lines tested (see PFA (−)). In contrast, when the immunoblot membrane was fixed with PFA, endogenous α-syn was strongly detected in SH-SY5Y and Daoy cells (see PFA (+)).

**Figure 5:**
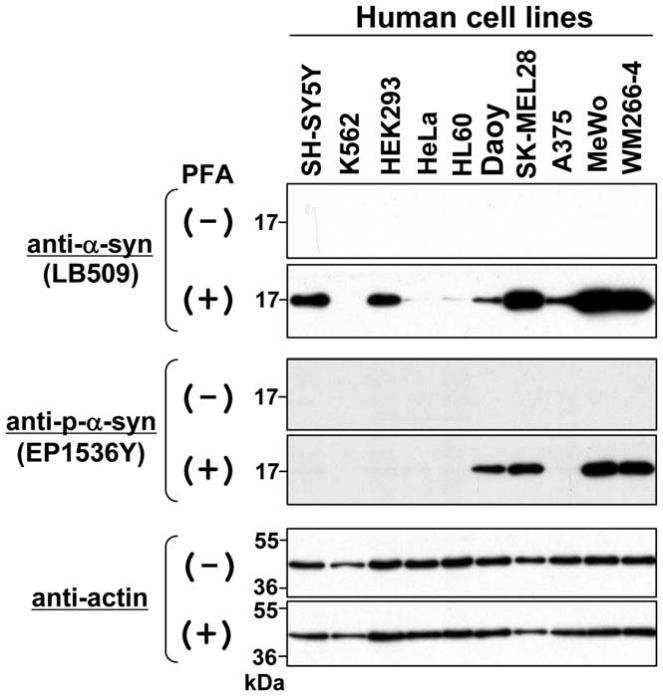
Immunoblotting of endogenous α-syn phosphorylated at Ser-129 in various human cell lines. Total cell lysates were prepared from 10 human cell lines: SH-SY5Y (neuroblastoma), K562 (erythroleukemia), HEK293 (human embryonic kidney cell), HeLa (cervical adenocarcinoma), HL60 (promyelocytic leukemia), Daoy (medulloblastoma), SK-MEL28 (malignant melanoma), A375 (malignant melanoma), MeWo (malignant melanoma), and WM266-4 (malignant melanoma). The protein samples (∼10 µg) were loaded onto a 15% SDS-polyacrylamide gel and electrophoresed, followed by Western transfer onto a PVDF membrane. The membrane was then fixed with or without 0.4% PFA for 30 min. Afterwards, the membrane was blocked and incubated with a primary antibody (anti-α-syn antibody LB509, anti-phospho-α-syn antibody EP1536Y, or anti-actin antibody) and a secondary antibody conjugated with horseradish peroxidase. Protein bands on the membrane were detected by ECL-Plus detection system. Symbols (+) and (–) indicate presence and absence of membrane fixation with 0.4% PFA, respectively. Molecular size markers are shown in kilodaltons (kDa).

As described above, PFA fixation of blotted membranes dramatically improved the detection of endogenous α-syn in many cell lines. This dramatic improvement raised the question as to whether phosphorylated α-syn can be detected in these cell lines. In Lewy bodies of patients with PD, α-syn is predominantly phosphorylated at Ser-129 (S129) [9], [14], suggesting the importance of this phosphorylation in disease pathogenesis. Therefore, the S129 phosphorylation should be investigated using neuronal cells. Although we have had difficulty in its detection by a conventional immunoblotting method, we may detect S129-phosphorylated α-syn by immunoblotting using PFA fixation. To test this possibility, we performed immunoblotting using protein samples in which α-syn was detectable in Figs. 2, 3, and 4. As shown in the middle panels of Fig. 5, EP1536Y antibody detected S129-phosphorylated α-syn in Daoy, SK-MEL28, MeWo, and WM266-4 cell lines with membrane fixation, but not in other cell lines including SH-SY5Y (see PFA (+)). As expected, the phosphorylated α-syn was undetectable by EP1536Y on the blotted membrane without fixation (see PFA (−)).

**Figure 6:**
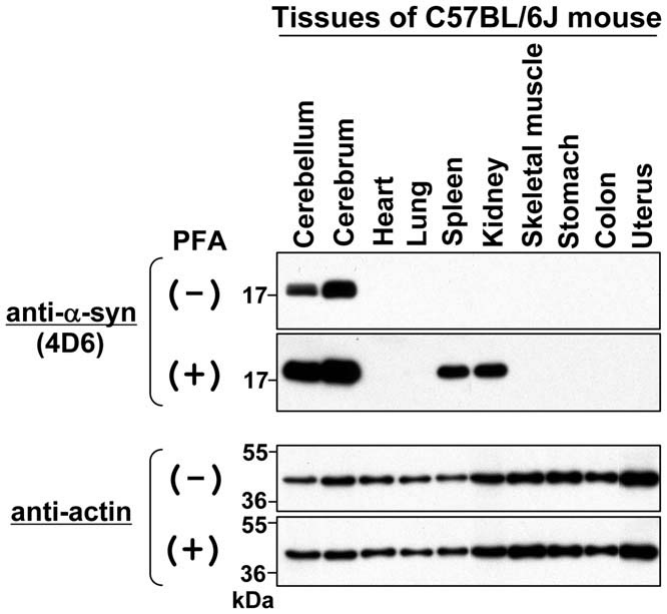
Immunoblotting of endogenous α-syn in various mouse tissues. Tissue lysates were prepared from C57BL/6J mice. The protein samples (∼10 µg) were loaded onto a 15% SDS-polyacrylamide gel and electrophoresed, followed by Western transfer onto a PVDF membrane. The membrane was then fixed with or without 0.4% PFA for 30 min. Afterwards, the membrane was blocked and incubated with a primary antibody (anti-α-syn antibody 4D6 or anti-actin antibody) and a secondary antibody conjugated with horseradish peroxidase. Protein bands on the membrane were detected by ECL-Plus detection system. Symbols (+) and (−) indicate presence and absence of membrane fixation with 0.4% PFA, respectively. Molecular size markers are shown in kilodaltons (kDa).

Finally, we performed immunoblotting using various mouse tissues. As shown in Fig. 6, endogenous α-syn was detected only in the cerebellum and cerebrum by a conventional method (PFA (−)), whereas it was detected in the spleen and kidney in addition to the cerebellum and cerebrum on a PFA-fixed membrane (see PFA (+)).

### Evidence for Specific Immunodetection of α-Syn on a PFA-fixed Membrane

**Figure 7:**
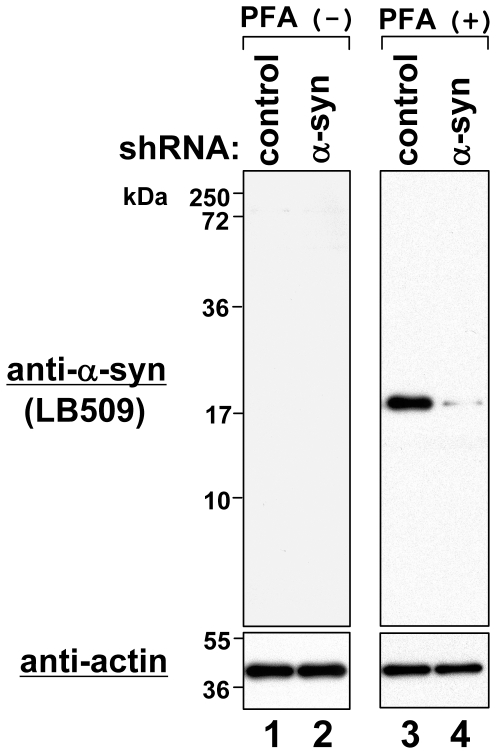
Detection of α-syn knockdown effect by immunoblotting using conventional method and improved method. In SK-MEL28 cells, shRNA of control or α-syn was expressed by lentivirus infection. Total cell lysates were loaded onto a 15% polyacrylamide gel, followed by immunoblotting using anti-α-syn antibody LB509 and anti-actin antibody. PFA (+) and (−) indicate presence and absence of membrane fixation with 0.4% PFA, respectively. Molecular size markers are shown in kilodaltons (kDa).

In immunostaining with PFA fixation, tissues and cultured cells are fixed with 4% PFA. Sometimes, however, this fixation generates non-specific staining. Therefore, although the membrane was fixed with highly diluted PFA (0.4%) in our immunoblotting, it was possible that the band on the fixed membrane was non-specific. To rule out this possibility, we used SK-MEL28 cells in which endogenous α-syn was knocked down by shRNA. As control, we used SK-MEL28 cells expressing control shRNA. In brief, we prepared total cell lysates from these cells and examined the expression levels of α-syn by immunoblotting with or without membrane fixation. As shown in Fig. 7, α-syn bands were almost undetectable by anti-α-syn antibody LB509 when the membrane was not fixed with PFA (lanes 1 and 2). However, only a 17-kDa band was strongly detected in the control SK-MEL28 cells when the membrane was fixed with PFA (lane 3). As expected, this band almost disappeared in the sample of α-syn-knockdown SK-MEL28 cells (lane 4). These results suggest that the 17-kDa band detected on the PFA-fixed membrane represents endogenous α-syn.

### Detachment of α-Syn Monomers from Western-transferred Membrane

As shown above, detection of endogenous α-syn is dramatically improved by mild fixation of the blotted membrane with PFA. There are two possible explanations for this improvement in detection. One explanation is that the PFA treatment unmasks epitope(s) of α-syn. The other explanation is that the PFA fixation effectively immobilizes α-syn on the blotted membrane. In other words, α-syn easily detaches from the blotted membrane without PFA fixation. The first explanation is unlikely to be correct because we used three different monoclonal antibodies (4D6, LB509, and EP1536Y) that react with distinct epitopes. It is most unlikely that all three epitopes are unmasked by the PFA treatment. The second explanation appears to be more likely. To prove this, we used the same samples shown in Fig. 2 (total cell lysates of SK-MEL28, SH-SY5Y, and BJAB) and performed immunoblotting using three different protocols (A, B, and C). Briefly, in Protocol A, the conventional immunoblotting method (without PFA fixation) was used. In Protocol B, the membrane was fixed with 0.4% PFA for 30 min just after Western transfer. In Protocol C, after Western transfer, the membrane was incubated for 3 h 45 min in TBS-T buffer (to match the incubation time of Protocol A) and then fixed with 0.4% PFA for 30 min. Afterwards, these membranes were blocked and incubated with a primary antibody (anti-α-syn 4D6, LB509, or anti-actin) and a secondary antibody conjugated with horseradish peroxidase. Protein bands on the membranes were detected by ECL-Plus detection system.

**Figure 8:**
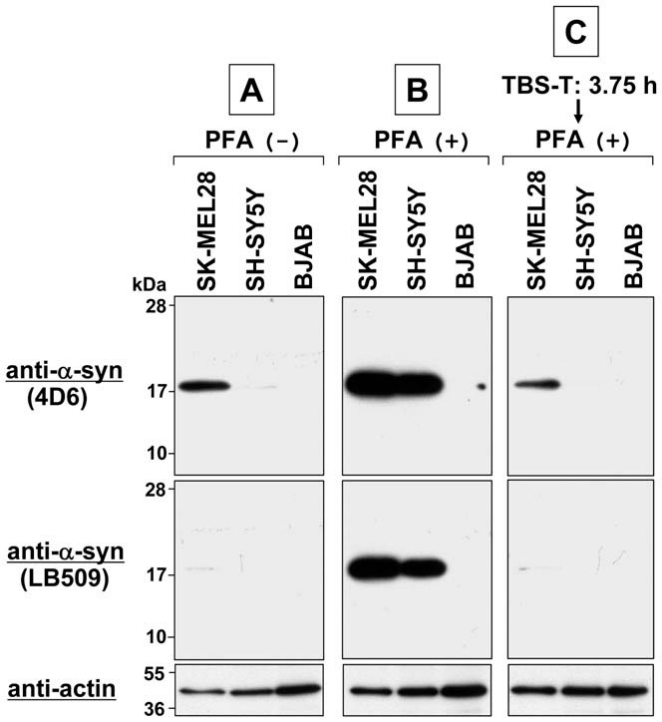
Detachment of α-syn monomers from Western-transferred membrane. Total cell lysates were prepared from SK-MEL28 (positive control), SH-SY5Y (positive control), and BJAB (negative control) cells. The protein samples (∼10 µg) were loaded onto a 15% SDS-polyacrylamide gel and electrophoresed, followed by Western transfer onto a PVDF membrane. The membrane was then processed using three different protocols. In Protocol **A**, the conventional immunoblotting method (without PFA fixation) was used. In Protocol **B**, the membrane was fixed with 0.4% PFA for 30 min just after Western transfer. In Protocol **C**, the membrane was incubated for 3 h 45 min (3.75 h) in TBS-T buffer after Western transfer and then fixed with 0.4% PFA for 30 min. Afterwards, these membranes were blocked and incubated with a primary antibody (anti-α-syn 4D6, LB509, or anti-actin) and a secondary antibody conjugated with horseradish peroxidase. Protein bands on the membranes were detected by ECL-Plus detection system. Molecular size markers are shown in kilodaltons (kDa).

As shown in Fig. 8, when Protocol A and Protocol B were used, we had the same results as demonstrated in Fig. 2. In brief, endogenous α-syn was weakly detected in positive control samples when the conventional immunoblotting method (Protocol A) was used. In contrast, the detection of endogenous α-syn was dramatically improved when the blotted membrane was fixed with PFA (Protocol B). Importantly, when the blotted membrane was incubated for 3 h 45 min in TBS-T buffer after Western transfer (Protocol C), the subsequent PFA fixation was not effective for the detection of endogenous α-syn. These results suggest that α-syn monomers detach from the blotted membrane during incubation when not fixed with PFA.

### Immunoblotting of α-Syn using Nitrocellulose Membranes

**Figure 9:**
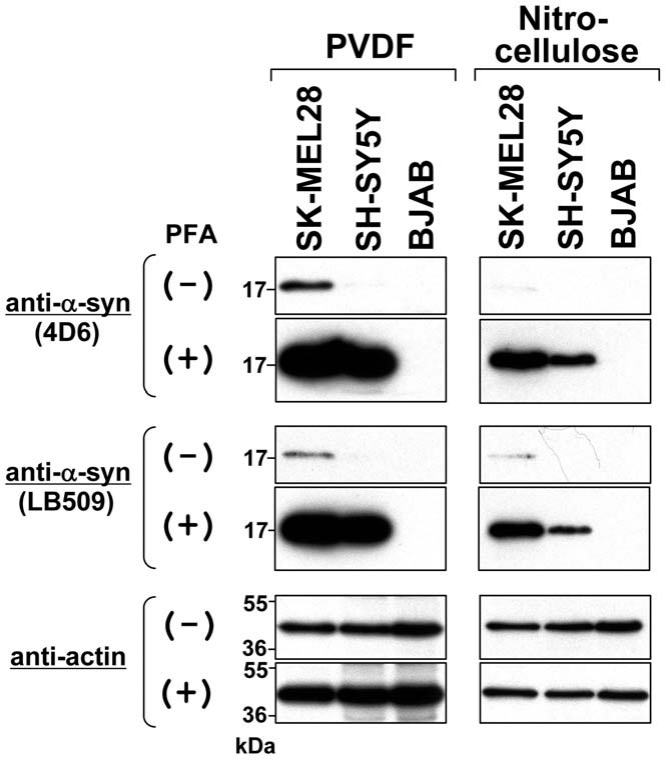
Comparison of nitrocellulose and PVDF membranes in immunoblotting of α-syn. Immunoblotting was performed using a PVDF membrane (left panels) and a nitrocellulose membrane (right panels). Briefly, total cell lysates were prepared from SK-MEL28 (positive control), SH-SY5Y (positive control), and BJAB (negative control). The protein samples (∼10 µg) were loaded onto a 15% SDS-polyacrylamide gel and electrophoresed, followed by Western transfer onto a PVDF or nitrocellulose membrane. The membrane was then fixed with or without 0.4% PFA for 30 min. Afterwards, the membrane was blocked and incubated with a primary antibody (anti-α-syn 4D6, LB509, or anti-actin) and a secondary antibody conjugated with horseradish peroxidase. Protein bands on the membrane were detected by ECL-Plus detection system. Symbols (+) and (−) indicate presence and absence of membrane fixation with 0.4% PFA, respectively. Molecular size markers are shown in kilodaltons (kDa).

PVDF and nitrocellulose are the two membrane types most commonly used in immunoblotting applications. In the immunoblotting experiments described above, we used PVDF membranes because it was previously reported that they show better protein retention [15]. However, there was a possibility that α-syn is detected more strongly on nitrocellulose membranes. To test this possibility, we performed immunoblotting using both PVDF and nitrocellulose membranes to detect endogenous α-syn. Specifically, total cell lysates were prepared from SK-MEL28 (positive control), SH-SY5Y (positive control), and BJAB (negative control). The protein samples were electrophoresed, followed by Western transfer onto a PVDF or nitrocellulose membrane. The membrane was then fixed with or without 0.4% PFA. Afterwards, endogenous α-syn was detected using anti-α-syn antibodies 4D6 and LB509. As shown in Fig. 9, PFA fixation dramatically improved detection of endogenous α-syn on both PVDF membranes (left panels) and nitrocellulose membranes (right panels). Importantly, the α-syn immunoblotting on PVDF membranes showed better detection than that on nitrocellulose membranes, probably due to better protein retention.

### Detection of Monomers and Aggregates of Recombinant α-Syn on a PFA-fixed Membrane

**Figure 10:**
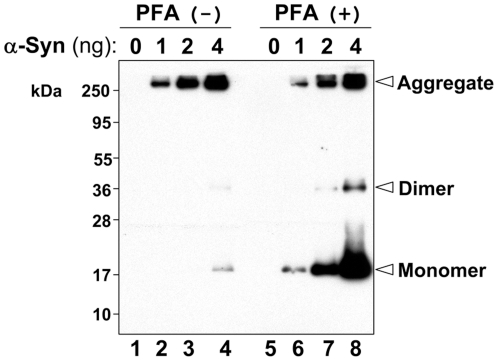
Detection of recombinant α-syn species by immunoblotting using conventional method and improved method. Indicated amounts of recombinant proteins were separated on a 12% SDS-polyacrylamide gel and transferred onto a PVDF membrane, followed by membrane fixation with or without 0.4% PFA. Afterwards, the membrane was blocked with skim milk and sequentially incubated with anti-α-syn antibody 4D6 and a secondary antibody. Finally, bands of recombinant α-syn species were detected by ECL-plus detection system. PFA (+) and (−) indicate presence and absence of membrane fixation with 0.4% PFA, respectively. Molecular size markers are shown in kilodaltons (kDa).

α-Syn tends to form aggregates *in vivo* and *in vitro* [2]. To investigate how aggregates and monomers of α-syn are detected on a PFA-fixed membrane, we performed immunoblotting using recombinant human α-syn generated and purified from bacteria. As shown in Fig. 10, when the membrane was not fixed with PFA, α-syn monomers were not clearly detected, whereas its aggregates with high molecular weight were strongly detected (lanes 1–4). In contrast, when the membrane was fixed with PFA, α-syn monomers were strongly detected in a loading-amount-dependent manner (lanes 5–8). In lanes 7 and 8, its dimers were also detected. Importantly, its aggregates on a PFA-fixed membrane (lanes 5-8) were detected at the same strength as detected on an unfixed membrane (lanes 1–4). These results suggest that monomers and dimers of α-syn were detached from the unfixed membrane during the experimental procedure (lanes 1-4). However, the detachment appears to be prevented by the PFA fixation (lanes 5–8). α-Syn aggregates do not seem to be affected seriously by the detachment problem (lanes 1-4 vs. lanes 5–8).

## Discussion

α-Syn is a key molecule in understanding the development of α-synucleinopathies such as PD, because α-synucleinopathies are caused by accumulation of α-syn that escapes from proteolytic cellular clearance [2]. To elucidate the pathogenesis of α-synucleinopathies, we must use neuronal cell lines to investigate endogenous α-syn. However, it has been almost undetectable by a conventional immunoblotting method in many human cell lines including neuronal cell lines such as SH-SY5Y [11]. This difficulty in detection of endogenous α-syn has largely restricted progress in α-synucleinopathy research. In this study, we modified the immunoblotting method by fixing blotted membranes with PFA, resulting in dramatic improvement in detection of endogenous α-syn in human cell lines including SH-SY5Y.

When a conventional immunoblotting method is used, why is there such difficulty in detection of endogenous α-syn? A clue to answer this question exists in results of our experiments using recombinant α-syn that contains monomers and aggregates. Specifically, our results indicate that α-syn monomers easily detach from blotted membranes, whereas α-syn aggregates are more stably immobilized on the blotted membranes. On the basis of these results, we have concluded that the difficulty in detection of endogenous α-syn results from the detachment tendency of α-syn monomers, and is not due to the anti-α-syn antibodies or sensitivity of immunodetection system. Importantly, the detachment of α-syn can easily be prevented by the PFA fixation of blotted membranes.

In α-synucleinopathies, α-syn accumulates and forms aggregates that are toxic to neurons, leading to neurodegeneration [2]. Therefore, many researchers have extensively investigated α-syn aggregates. In these studies, a conventional immunoblotting method has frequently been used to compare the expression levels of the aggregates with those of the monomers. However, our results imply that, although α-syn aggregates have correctly been detected in such studies, α-syn monomers have been detected at much lower levels than the actual expression levels. For example, if we analyze the recombinant α-syn purchased from Sigma by a conventional immunoblotting method, we should conclude that the majority of α-syn is aggregated (see lane 4, Fig. 10). But this interpretation is not correct. In point of fact, α-syn aggregates are much less than its monomers (see lane 8, Fig. 10). Thus, it is possible that we have overestimated the extent of aggregate formation of α-syn in α-synucleinopathy research. However, we are now able to avoid this detection problem by fixing the blotted membranes with PFA. This method may be very beneficial to studies of α-syn aggregates as well as monomers.

α-Syn is predominantly phosphorylated at S129 in Lewy bodies of PD patients [9], [14] and in Lewy body-like inclusions of transgenic mice expressing human mutant A53T α-syn [16], suggesting the importance of this phosphorylation in disease pathogenesis. However, α-syn phosphorylated at S129 constitutes only a very small fraction of soluble α-syn in the neuron [9], [14], which has been a barrier to research of S129-phosphorylated α-syn. In this study, using our modified immunoblotting method, we demonstrated that S129-phosphorylated α-syn is clearly detectable in some human cell lines. Thus, our method may also enhance research of S129-phosphorylated α-syn.

In conclusion, the PFA fixation of blotted membranes allows us to easily detect α-syn monomers (even their phosphorylated form) in cell lines and mouse tissues. This innovative modification in immunoblotting for α-syn will enhance progress in α-syn biology. In α-synucleinopathy research, however, α-syn in samples of human patients and PD model mice might act differently than α-syn in the samples presented here. Further studies will be required to apply our modified immunoblotting method to such pathological conditions.

## References

[1] Maroteaux L, Campanelli JT, Scheller RH (1988). Synuclein: a neuron-specific protein localized to the nucleus and presynaptic nerve terminal.. J Neurosci.

[2] Auluck PK, Caraveo G, Lindquist S (2010). α-Synuclein: membrane interactions and toxicity in Parkinson's disease.. Annu Rev Cell Dev Biol.

[3] Bennett MC (2005). The role of α-synuclein in neurodegenerative diseases.. Pharmacol Ther.

[4] McFarland MA, Ellis CE, Markey SP, Nussbaum RL (2008). Proteomics analysis identifies phosphorylation-dependent α-synuclein protein interactions.. Mol Cell Proteomics.

[5] Polymeropoulos MH, Lavedan C, Leroy E, Ide SE, Dehejia A (1997). Mutation in the α-synuclein gene identified in families with Parkinson's disease.. Science.

[6] Kruger R, Kuhn W, Muller T, Woitalla D, Graeber M (1998). Ala30Pro mutation in the gene encoding α-synuclein in Parkinson's disease.. Nat Genet.

[7] Singleton AB, Farrer M, Johnson J, Singleton A, Hague S (2003). α-Synuclein locus triplication causes Parkinson's disease.. Science.

[8] Zarranz JJ, Alegre J, Gomez-Esteban JC, Lezcano E, Ros R (2004). The new mutation, E46K, of α-synuclein causes Parkinson and Lewy body dementia.. Ann Neurol.

[9] Fujiwara H, Hasegawa M, Dohmae N, Kawashima A, Masliah E (2002). α-Synuclein is phosphorylated in synucleinopathy lesions.. Nat Cell Biol.

[10] Tanji K, Tanaka T, Mori F, Kito K, Takahashi H (2006). NUB1 suppresses the formation of Lewy body-like inclusions by proteasomal degradation of synphilin-1.. Am J Pathol.

[11] Matsuo Y, Kamitani T (2010). Parkinson's disease-related protein, α-synuclein, in malignant melanoma.. PLoS One.

[12] Suzuki Y, Takeda Y, Ikuta T (2008). Immunoblotting conditions for human hemoglobin chains.. Anal Biochem.

[13] Jakes R, Crowther RA, Lee VM, Trojanowski JQ, Iwatsubo T (1999). Epitope mapping of LB509, a monoclonal antibody directed against human α-synuclein.. Neurosci Lett.

[14] Anderson JP, Walker DE, Goldstein JM, de Laat R, Banducci K (2006). Phosphorylation of Ser-129 is the dominant pathological modification of α-synuclein in familial and sporadic Lewy body disease.. J Biol Chem.

[15] Lauritzen E, Pluskal M (1988). Improved HIV antiglycoprotein antibody detection by immunoblotting on a hydrophobic membrane.. J Acquir Immune Defic Syndr.

[16] Wakamatsu M, Ishii A, Ukai Y, Sakagami J, Iwata S (2007). Accumulation of phosphorylated α-synuclein in dopaminergic neurons of transgenic mice that express human α-synuclein.. J Neurosci Res.

